# Bystander T cells in human immune responses to dengue antigens

**DOI:** 10.1186/1471-2172-11-47

**Published:** 2010-09-20

**Authors:** Duangchan Suwannasaen, Arunrat Romphruk, Chanvit Leelayuwat, Ganjana Lertmemongkolchai

**Affiliations:** 1 The Centre for Research and Development of Medical Diagnostic Laboratories, Faculty of Associated Medical Sciences, Khon Kaen University, Khon Kaen, Thailand

## Abstract

**Background:**

Previous studies of T cell activation in dengue infection have focused on restriction of specific T cell receptors (TCRs) and classical MHC molecules. However, bystander T cell activation, which is TCR independent, occurs via cytokines in other viral infections, both in vitro and in vivo, and enables T cells to bypass certain control checkpoints. Moreover, clinical and pathological evidence has pointed to cytokines as the mediators of dengue disease severity. Therefore, we investigated bystander T cell induction by dengue viral antigen.

**Results:**

Whole blood samples from 55 Thai schoolchildren aged 13-14 years were assayed for in vitro interferon-gamma (IFN-γ) induction in response to inactivated dengue serotype 2 antigen (Den2). The contribution of TCR-dependent and independent pathways was tested by treatment with cyclosporin A (CsA), which inhibits TCR-dependent activation of T cells. ELISA results revealed that approximately 72% of IFN-γ production occurred via the TCR-dependent pathway. The major IFN-γ sources were natural killer (NK) (mean ± SE = 55.2 ± 3.3), CD4^+^T (24.5 ± 3.3) and CD8^+^T cells (17.9 ± 1.5), respectively, as demonstrated by four-color flow cytometry. Interestingly, in addition to these cells, we found CsA-resistant IFN-γ producing T cells (CD4^+^T = 26.9 ± 3.6% and CD8^+^T = 20.3 ± 2.1%) implying the existence of activated bystander T cells in response to dengue antigen in vitro. These bystander CD4^+ ^and CD8^+^T cells had similar kinetics to NK cells, appeared after 12 h and were inhibited by anti-IL-12 neutralization indicating cytokine involvement.

**Conclusions:**

This study described immune cell profiles and highlighted bystander T cell activation in response to dengue viral antigens of healthy people in an endemic area. Further studies on bystander T cell activation in dengue viral infection may reveal the immune mechanisms that protect or enhance pathogenesis of secondary dengue infection.

## Background

T cell mediated production of cytokines, such as TNF-alpha, interferon-gamma (IFN-γ) and interleukin (IL)-10, has been reported to influence the severity of dengue infection [1-5]. The mechanisms of T cell activation are mostly focused on the classical pathway, that is activation via binding of specific T cell receptors (TCRs) and MHC molecules [6,7]. However, T cells may also be activated after stimulation by 'bystander' or TCR-independent signaling, for example by cytokines or novel activating receptors [8-12]. Bystander T cell activation has been demonstrated in models of viral infection such as herpes simplex virus, LCMV and HIV leading to proliferation of memory T cells and subsequent production of cytokines, which can induce protection or pathology [9,11,13]. In addition to virus infection, our studies have identified IFN-γ producing bystander CD8^+^T cells in response to intracellular bacteria and showed that these T cells produced IFN-γ within 24 h [10]. The mechanism of bystander IFN-γ activation depends on pro-inflammatory cytokines, mainly IL-12 and IL-18 [14].

Dengue viral infection is the cause of dengue fever (DF) and the more severe dengue hemorrhagic fever (DHF) [15]. Secondary infection by dengue virus of different serotypes to the primary infection in children aged less than 15 years is significantly associated with severe DHF [16,17]. Previous studies have revealed that a storm of pro-inflammatory cytokines is released during acute infection [18]. These observations suggest that bystander T cell activation might possibly occur in dengue infection.

In this study, we aimed to investigate the existence of bystander T cell activation in healthy children living in endemic areas who might be vulnerable to reinfection with dengue virus and at risk of developing DHF [16]. We examined IFN-γ production, which is the established indicator for bystander T cell activation [10], after restimulating with inactivated dengue viral antigens in vitro. Bystander T cell activity was demonstrated by resistance to cyclosporin A (CsA), which is a substance known to inhibit T cell activation via the TCR-dependent pathway [9,19,20] In addition, we described the kinetics of bystander T cells and cytokines involved in IFN-γ-derived T cell activation. The description of immune profiles in this study highlights bystander activation in natural DV infection of healthy people in an endemic area and emphasizes that the immune responses to dengue virus are more complex than anticipated.

## Results

### Healthy Thai schoolchildren could produce IFN-γ in response to inactivated dengue virus serotype 2 in vitro

IFN-γ was selected for determination as a marker of bystander T cell function in this study. The mechanisms of IFN-γ production, triggered via TCR-dependent or independent pathways, were investigated by treatment with CsA, which is known to inhibit T cells via a TCR-dependent pathway. Blood samples from 55 healthy Thai schoolchildren aged 13-14 years were co-cultured with control stimulators (medium, phytohemagglutinin (PHA) and a combination of IL-12 plus IL-15 cytokines) or Den2 in the absence or presence of CsA for 48 h. The cultured supernatants were assayed for IFN-γ by sandwich ELISA.

**Figure 1:**
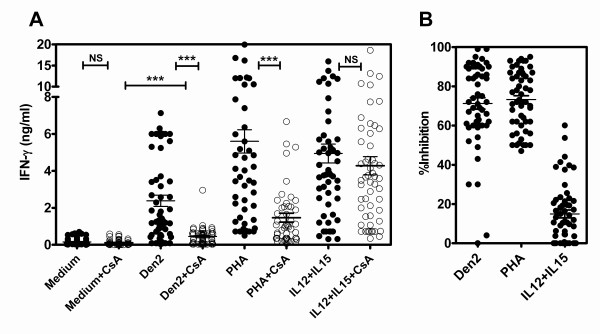
**IFN-γ induction by inactivated dengue virus serotype 2 of 55 healthy Thai children aged 13-14 years**. Whole blood samples containing 9 × 10^5 ^lymphocytes/ml were co-cultured with medium alone, Den2, PHA and IL-12 plus IL-15 in the presence or absence of 0.3 μg/ml cyclosporin A (CsA) for 48 h. The cultured supernatants were assayed for IFN-γ by sandwich ELISA (A). Percentages of IFN-γ inhibition as calculated by CsA treatment (B). *** represents the statistically significant difference between the two groups (*P *< 0.0001, Mann Whitney test; NS represents non significant).

The positive control stimulators induced significantly higher IFN-γ than the medium control (Figure 1A). In this study, the reduction of IFN-γ levels (as inhibited by CsA) was defined as CsA sensitive or IFN-γ triggered via the TCR-dependent pathway, and CsA resistant levels were defined as IFN-γ triggered via the TCR-independent pathway. As expected, in the control stimulation, IFN-γ induced by PHA, but not IL-12 plus IL-15, was significantly inhibited by CsA treatment (*P *< 0.001, Mann Whitney test) indicating that CsA sufficiently inhibited IFN-γ triggering via the TCR-dependent but not the cytokine receptor dependent pathway. Interestingly, IFN-γ induced by Den2 stimulation was also significantly inhibited by CsA (*P *< 0.001, Mann Whitney test). Moreover, the remaining IFN-γ levels after adding CsA were also significantly higher than medium plus CsA control (*P *< 0.001, Mann Whitney test).

Inhibition by CsA treatment in response to Den2, PHA and IL-12 plus IL-15 stimulation was 71.2 ± 2.9% (mean ± SE), 73.2 ± 2.0% and 12.1 ± 2.7%, respectively (Figure 1B). These results implied that IFN-γ induction by Den2 was mainly activated by the TCR-dependent pathway (approximately 70%) and to a lesser degree by TCR-independent signaling (approximately 30%).

To validate whether CsA does indeed inhibit all TCR-stimulated IFN-γ activation, a MHC class I-restricted T cell epitope control of pooled peptides (CEF) of cytomegalovirus, Epstein Barr virus and influenza virus (Mabtech, AB, Sweden) was included in a small study with five whole blood samples from healthy school children stimulated with medium control, Den2, PHA and CEF in the presence or absence of 0.3 μg/ml of CsA. The results clearly showed that CsA could completely inhibit IFN-γ induction by CEF stimulation, but it partially inhibited IFN-γ induced by Den2. These results confirmed that CsA could inhibit all IFN-γ production from TCR-dependent activation (See Additional file 1, Figure S1).

Moreover, we have previously titrated CsA concentration to test the ability of IFN-γ inhibition. IFN-γ induced by PHA, which is the strongest IFN-γ stimulator, was inhibited by CsA in a dose-dependent manner and maximum inhibition occurred at 0.3 μg/ml of CsA and remained constant at higher doses. This effect could be detected at time points 4 and 42 h after incubation (See Additional file 1, Figure S2). These results revealed that CsA at this optimal concentration was able to inhibit T activation, via TCR-dependent activation, in both polyclonal and antigen-specific T cell activation.

### Identification of IFN-γ^+ ^cells that responded to Den2

To identify the types of immune cells that responded to dengue virus in the presence or absence of CsA, the intracellular sources of IFN-γ were characterized by four-color flow cytometry. Eighteen children who had strong IFN-γ responsiveness were followed up to determine the IFN-γ producing cells. The collected whole blood samples containing 9 × 10^5 ^lymphocytes/ml were co-cultured with medium and 18 HA (hemagglutination) units Den2 for 24 h in the absence or presence of CsA prior to cell staining.

**Figure 2:**
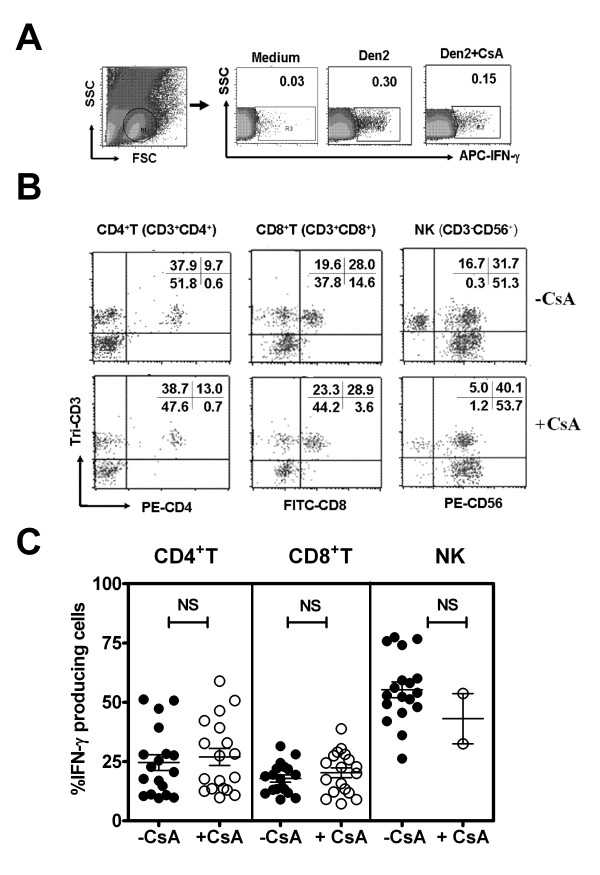
**Identification of IFN-γ producing cells using flow cytometry**. Cells cultured with medium, Den2 with or without CsA at 24 h were stained with tri-CD3, FITC-CD8 and PE-CD4 or PE-CD56 and APC-IFN-**γ**. Gating profile of small lymphocyte (R1) and IFN-γ^+ ^cell (R3) from one representative sample (A). Gated IFN-γ producing cells were analyzed with cell surface marker profiles (B). The distribution of IFN-γ producing CD4^+^, CD8^+^T and NK cells from 18 samples were plotted and compared between absence (-CsA) and presence of CsA (+CsA) (C) by Mann Whitney test (NS represents non significant).

In flow cytometric analysis, total cell populations were analyzed by side scatter and forward scatter plotting. Then, the small lymphocyte population was first gated and shown as region R1. Total IFN-γ^+ ^cells were then gated and shown as region R3 (Figure 2A). The types of IFN-γ^+ ^cells were then analyzed by the combination of tri-CD3, FITC-CD8 and PE-CD4 or PE-CD56 and compared in the presence and absence of CsA (Figure 2B).

The results revealed that Den2 stimulation induced total %IFN-γ^+ ^cells significantly higher than medium controls, and approximately 43% (43.2 ± 6.0) were inhibited by CsA (data not shown). Adding CsA resulted in the reduction of total %IFN-γ+ cells, and the proportions of IFN-γ^+ ^lymphocyte subsets were slightly changed (none statistically significant) by the decrease of natural killer (NK) and increase of T cells. Moreover, the results clearly demonstrated that some CD4^+ ^and CD8^+^T cells resisted the effect of CsA, suggesting that dengue virus induces bystander T cell activation in vitro (Figure 2B).

The IFN-γ producing immune cell types in response to Den2 stimulation consist of NK cells (mean ± SE = 55.2 ± 3.3%), CD4^+^T (24.5 ± 3.3%) and CD8^+^T (17.9 ± 1.5%) cells, respectively. Interestingly, the distribution of CsA resistant or bystander CD4^+^T cells ranged from 10 to 59% (mean ± SE = 26.9 ± 3.1%) and CD8^+^T cells ranged from 7 to 39% (20.3 ± 2.1%), respectively (Figure 2C). Of note, due to the shortage of blood samples in this experiment, only 2 of 18 blood samples were studied in the presence of CsA and stained for NK cells. These results indicated that bystander (both CD4^+ ^and CD8^+^) T cells and NK cells could contribute equally to the production of IFN-γ in responses to dengue virus in vitro.

The additional analysis of NK cell surface markers clearly demonstrated that CD3-CD56+CD16+ cells were also sources of IFN-γ (23.9 ± 7.1% of NK cells) (data not shown).

### Kinetics of bystander T and NK cell activation

**Figure 3:**
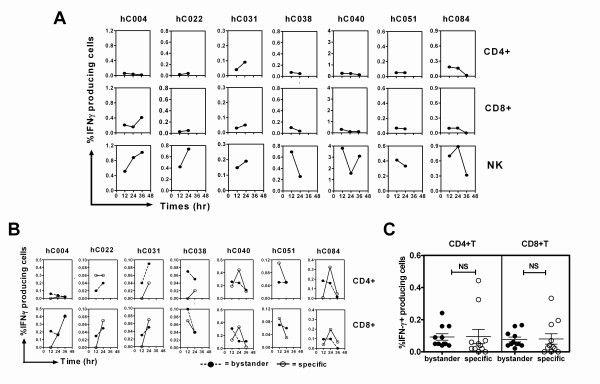
**Kinetics of TCR signaling-specific vs. bystander IFN-γ producing CD4**^+^**T and CD8**^+^**T cell subsets**. The cultured cells from seven representative samples were cultured with Den2 and analyzed the frequencies of IFN-γ^+ ^CD4^+ ^or CD8^+ ^T cells in the presence of CsA (bystander T cells) or NK cells at 12, 24 and 36 h (A). The kinetics of specific vs. bystander CD4^+ ^or CD8^+ ^T cells (B). The distributions of specific vs. bystander CD4^+ ^or CD8^+ ^cells activated with Den2 for 24 h from 11 samples were compared using the Mann Whitney test (NS represents non significant). The isotype controls were also stained and showed the background of % IFN-γ producing cells less than 0.01%.

To describe characteristic of activated bystander T cells, the kinetics of IFN-γ production of those bystander T cells were compared with innate NK cells and activated specific T cells. Blood samples from seven schoolchildren were studied after 12, 24 and 36 h stimulation (details as described in the above section). The results revealed that IFN-γ^+ ^cells could be detected as early as 12 h and most of seven samples showed similar kinetics of bystander CD4^+^, CD8^+^T and NK cells (Figure 3A). One particular sample (hC004) showed a CD8+T predominant response with almost no CD4^+ ^T cells. These results demonstrated the similar characteristics of bystander T cells in producing IFN-γ in addition to NK cells in response to dengue virus. In contrast to bystander CD4^+ ^and CD8^+^T cells, specific CD4^+ ^and/or CD8^+^T cells, were not detected as early as 12 h in five of seven samples (hC004, hC022, hC031, hC038 and hC084) (Figure 3B). The proportion of bystander and specific T cells from 18 samples, however, was equal after 24 h culture (Figure 3C). Taken together, these results indicated that innate immune cells (NK cells and bystander T cells) were rapidly induced in the early response and were followed by specific T cells to produce IFN-γ in response to dengue virus.

### IL-12-dependent pathway mediated T cell to produce IFN-γ

Previously, proinflammatory cytokines such as IL-12, IL-15 and IL-18 have been reported to elicit bystander T cells to produce IFN-γ [10,14]. To confirm that anti-cytokine neutralizing antibodies are, in fact, capable of inhibiting the cytokines at the concentrations used, the whole blood samples from healthy donors were treated with three-fold serial concentration of anti-IL-12, anti-IL-15 and anti-IL-18 (at 0.3, 1 and 3 μg/ml, respectively) for 15 minutes before stimulation with heat-inactivated *Burkholderia pseudomallei*, a strong bystander or cytokine-dependent T cell inducer as shown previously by our group. After 48 h, the cultured supernatants were harvested and examined for IFN-γ level by ELISA. The results showed that all three anti-cytokine neutralizing antibodies could decrease IFN-γ induction by *B. pseudomallei *in a dose-dependent manner. Therefore, the concentration of these antibodies at 1 μg/ml was used in the following Den2 experiments (See Additional file 1, Figure S3).

**Figure 4:**
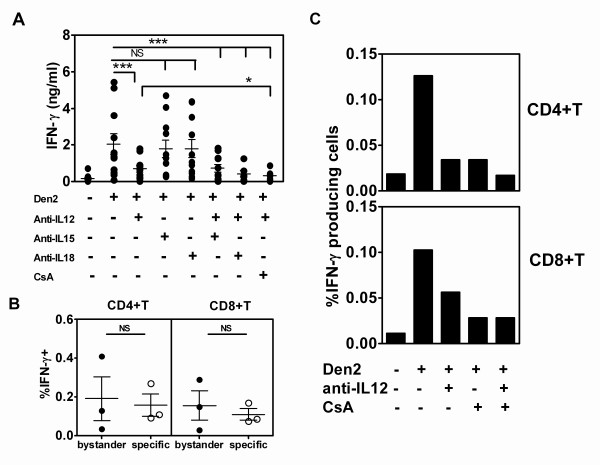
**Effects of pro-inflammatory cytokine neutralizing antibodies on IFN-γ production**. Whole blood samples collected from 11 healthy donors were treated with monoclonal antibodies to IL-12, IL-15, IL-18 or CsA alone or in combination before adding inactivated Den2 for 48 h. IFN-γ levels in all conditions were assayed and compared using the Wilcoxon matched pair test (***, * represent statistically significant by *P *< 0.001 and *P *< 0.05, respectively; NS represents non significant) (A). Distribution of IFN-γ producing bystander vs. specific induced by Den2 from three healthy adults were plotted and compared by using Mann Whitney test (B). IFN-γ production by whole blood cells from one representative healthy donor was induced by Den2 in the presence of 0.8 μg/ml anti-IL-12, CsA or a combination of anti-IL12 and CsA at the time point of 24 h. IFN-γ producing cells in each subpopulation were shown (C). ND represents not determined.

To investigate these cytokines in IFN-**γ **production in response to dengue virus, the effects of neutralizing antibodies to these cytokines were investigated. Eleven whole blood samples from healthy adult blood donors were studied by adding neutralizing monoclonal antibodies to human IL-12, IL-15 and IL-18, and testing the culture supernatants for IFN-γ by ELISA. The results revealed that adding anti-IL-12, but not anti-IL-15 nor anti-IL-18, resulted in the statistically significant reduction of IFN-γ in response to dengue virus stimulation. These results revealed that IFN-γ was mainly activated by the IL-12-dependent pathway. Additionally, IFN-γ was completely inhibited by the combination of anti-IL-12 and CsA treatment suggesting synergistic effects of IL-12-dependent and TCR-dependent pathways (Figure 4A). Three representative donors were then selected to characterize the intracellular sources of IFN-γ by flow cytometry using the protocol described above. The results showed that, as for the blood samples from children, the major IFN-γ producing cells were NK, CD4^+^T and CD8^+^T, respectively (data not shown). Additionally, the distribution of bystander vs. specific CD4^+ ^or CD8^+^T cells was not significantly different (Figure 4B). To determine effect of neutralizing monoclonal antibodies on the IFN-γ producing subpopulation, two representative samples were studied. Results showed that anti-IL-12 and CsA could decrease IFN-γ producing cells derived from both CD4^+ ^and CD8^+^T cells and the synergistic effect of the two pathways was also observed. CD4^+^T cells were equally sensitive to anti-IL-12 and CsA whereas CD8^+^T cells were less sensitive to anti-IL-12 than CsA (Figure 4C). In contrast to T cells, NK cells were more sensitive to anti-IL-12 than CsA, as suggested in one case where NK cell markers were included and approximately 34% of IFN-γ production from NK cells was inhibited by CsA, while anti-IL-12 was able to inhibit IFN-γ production up to 75%. These results confirmed that there were bystander T cells that could produce IFN-γ via an IL-12-dependent pathway in response to dengue infection.

## Discussion

In this study we first demonstrated that healthy Thai schoolchildren could produce IFN-γ in response to dengue virus serotype 2, which is the predominant serotype and the major cause of DHF [16]. Then, we highlighted the major finding that bystander CD4^+ ^T and CD8^+ ^T cells responding to dengue virus could account for approximately 50% of immune components producing IFN-γ via the TCR-independent pathway as demonstrated by their resistance to CsA (See Additional file 1, Figure S4 for the details of calculation). The contribution of these innate and/or adaptive immune components was demonstrated by the study of time kinetics, and the results clearly showed that the kinetics of bystander T cells were similar to those of NK cells and, in most cases, were in contrast to specific T cells. These results suggest a key function of innate immunity composed of NK and bystander T cells in rapid responses to control early dengue virus infection.

Determination of bystander T cell activation by using CsA treatment has been previously used by our group and others [9,21]. Moreover, the studies using neutralizing antibodies for IL-12, IL-15, IL-18 [14,19], or blocking antibodies for their receptors [21,22] has confirmed that bystander T cells and NK cells could be activated by these cytokines. Our studies clearly demonstrated that there were CD4^+ ^and CD8^+ ^T cells resistant to CsA treatment in healthy children's blood samples responding to dengue virus. These bystander T cells were strongly activated via an IL-12-dependent pathway as demonstrated by neutralization of anti-IL-12 but not anti-IL-15 or anti-IL-18. Additionally, no synergistic action between IL-12 and IL-15 or IL-18 could be observed suggesting that the activation of T cells via IL-12 alone was sufficient to produce IFN-γ. Paganin *et al*. have reported that IL-12 alone primes both CD4^+ ^and CD8^+^T clones for high IFN-γ production, and the principle mechanism is the ability to prime T cells during Th1 clonal expansion [23]. In addition to IL-12 alone, T cells also secrete IFN-γ in a TCR-independent manner after stimulation with the pro-inflammatory cytokines; IL-12, IL-15 and IL-18 or the synergistic actions of IL-12 and either IL-18 or IL-15 [24,25]. However, CsA could inhibit some IFN-γ producing NK cells. This unexpected effect of CsA on NK cells has also been demonstrated by Wang *et al*. The co-culture of peripheral blood NK cells subpopulations with CsA can decrease proliferation of CD56^dim ^NK cells (CD3^-^CD56^+^CD16^+^) but not CD56^bright ^NK cells (CD3^-^CD56^+^CD16^-^) [26]. Our studies have shown that CD56^dim ^NK cells produced IFN-γ in cell culture. Therefore, CsA might affect this NK cell subpopulation.

In addition, CD16^-^CD56^+ ^NK cells, CD3^-^CD56^+^CD16^+ ^NK cells, CD3^+^CD56^+ ^NKT cells, CD3^-^CD8^+ ^cells and CD3^-^CD4^+ ^cells were also found to produce IFN-γ in this study. Azeredo *et al*., have shown the increase of CD3^-^CD56^+^CD16^+ ^(NK cells), CD3^+^CD56^+ ^(CD56^+^T cells) during acute dengue infection [27]. Our findings supported the idea that those cells could respond by IFN-γ production against dengue virus at least in an IL-12-dependent manner. It has also been reported that NK cells control early dengue virus infection via antibody-dependent cellular cytotoxicity [28]. In light of recent evidence, the barrier between innate and adaptive immunity seems to be blurred [29]; our study offers the possibility of investigating this novel concept of host immunity in dengue infection.

One study of immune responses of volunteers who had received the live attenuated monovalent dengue vaccines reported that restimulating memory CD4^+ ^T cells in peripheral blood mononuclear cells with homologous inactivated dengue serotype resulted in the highest IFN-γ production whereas stimulation with heterologous serotypes resulted in the alteration of IFN-γ to TNF-alpha production [30]. Another study from the same group reported that dengue virus-reactive CD8^+^T cells showed differences in both proliferation and cytokine production responding to heterologous viral serotypes. Restimulation of these CD8^+ ^T cells with dengue serotype 3 variant of NS4b epitope 2423 and dengue 2 variant of NS4a epitope 2148 resulted in the greatest cytokine production [31]. Moreover, Mongkolsapaya *et al. *have reported that cross-reactive dengue specific CD8^+ ^T cells seemed to show suboptimal degranulation but also high IFN-γ and TNF-alpha production [1]. These results implied that specific T cell responses might lead to high levels of IFN-γ production whereas cross-reactive T cell responses alter cytokine profiles and contribute to disease severity. In respect of our studies, IFN-γ induced by Den1, 3 and 4 were also investigated. However, the IFN-γ induced by these antigens was much less than Den2 stimulation. These results might reflect the activation of Den2-specific T cells. However, relative contribution of specific vs. cross-reactive vs. bystander T cells to immunopathogenesis of dengue infection requires further investigation.

Nevertheless, the pre-existing immunity which protects or increases disease severity during secondary infection has not been well defined. The previous prospective study of Thai schoolchildren has revealed that the higher degree of activation of pre-existing IFN-γ responding to any of the four serotypes correlated with the subsequent hospitalization of children aged 7-10 years [5]. The IFN-γ induction in our study generally showed higher levels than the previous study of Mangada *et al*.; this could be due to the fact that all cellular functions in our study were performed in whole blood samples freshly collected within 4 h to avoid any decreased activities by the cryopreservation process of frozen samples. In addition, among 55 healthy school children in this study, 11 were seronegative and 44 were seropositive for IgG anti-dengue antibody. However, bystander IFN-γ production from these two populations was not different. Thus the degree of total or bystander IFN-γ production may be further studied as a potential predictor for DHF of sufficient severity to require hospitalization.

This study focused on bystander IFN-γ production in responses to inactivated dengue viral antigens in vitro using the same dengue viral preparation as for currently available commercial antibody assays. Unfortunately, a non-infected control from the same commercial source was not available for confirmation of antigen specific activation. Since it was prepared from suckling mouse brain, no animal laboratories could provide the control brain tissues without a proper license. Thus, IFN-γ induced by another source of inactivated antigens, cultured supernatants of C6/36 mosquito cells infected with dengue viral serotype 2 (16681 strain), was compared instead (See Additional file 1, Figure S5). Inactivated culture supernatants of mock-infected C6/36 cell lines showed no IFN-γ induction and the inactivated supernatants of Den2-infected C6/36 cells could stimulate cells to produce IFN-γ to higher levels than the medium control. Much less IFN-γ was induced by inactivated cultured supernatants than by mouse extracts. However, regression analysis of IFN-γ induced by two antigens showed that bystander but not specific IFN-γ levels were correlated (See Additional file 1, Figure S6). These results strongly supported the observation that dengue antigens could trigger bystander IFN-γ production in vitro.

## Conclusions

We have defined the bystander activation of T cells in responses to dengue infection, mediated by IL-12-dependent pathways existing in healthy Thai schoolchildren. These bystander T cells play a major contribute to the IFN-γ production in response to dengue virus in vitro. These immunological responses may be involved in immunopathogenesis of dengue hemorrhagic fever and provide basic information on immune cell profiles in children living in endemic areas with potential use for dengue vaccine development.

## Methods

### Subjects

The subjects were 55 healthy schoolchildren (17 females, 38 males, and aged 13-14 years) who were studying at the Secondary School, Phu Wat Pittayakom, Ubonrat District, Khon Kaen Province, Thailand. Blood samples were collected from each child to screen for IFN-γ production using ELISA. Follow-up blood samples were collected from 18 children for characterization of intracellular IFN-γ sources by four-color flow cytometry. None of the children had had any febrile episode in the seven days prior to collection of the whole blood samples. Twelve children had previously been hospitalized because of dengue infection. The protocol for the collection of whole blood samples has been reviewed and approved by Khon Kaen University Ethics Committee for Human Research (project number HE470510). All children and their parents signed the informed consent form before whole blood collection and the data in the consent forms were not disclosed. Additionally, 11 blood samples were collected from healthy adults to study cytokine neutralization. The samples were processed using the same protocol.

### Stimulators

#### Control stimulators

The negative control was cell culture medium (10% fetal bovine serum in RPMI). The positive controls for IFN-γ activation via the TCR-dependent pathway and cytokine activation were 2.5 μg/ml PHA (Roche, USA) and 0.1 ng/ml IL-12 plus IL-15, respectively.

#### Dengue viral antigens

Lyophilized mouse brain extracts containing dengue viral antigen serotype 1 (Den1, Hawaii strain), Den2 (Tr 1751 strain), Den3 (H 87 strain) and Den4 (H 241 strain) were purchased from the Department of Medical Science, Ministry of Public Health, Thailand for use in this study. These antigens were prepared by sucrose acetone extraction of mouse brains infected by dengue serotype 1-4 [32]. Preliminary studies revealed that 18 HA units of Den2 could induce the highest IFN-γ production compared to other serotypes. Thus this optimal antigen concentration was used in all experiments.

### Whole blood culture assay for IFN-γ induction

Whole blood samples (18 ml) were collected from subjects in heparinized tubes. Complete blood cells counts were performed using an automatic analyzer (Sysmex SF-3000, Germany). Lymphocyte numbers were adjusted to 9 × 10^5 ^cells/ml by diluting with culture medium (RPMI with 10% fetal bovine serum). The cells were freshly co-cultured with stimulators in 96-well culture plates and incubated in 5% CO_2 _at 37°C for 12, 24, 36, and 48 h, or as required.

To distinguish the IFN-γ production induced by TCR-dependent and TCR-independent pathways, CsA (Sigma, USA), a substance known to inhibit T cell activation via the TCR-dependent pathway, was added at 0.3 μg/ml which was the optimal concentration to inhibit IFN-γ production induced by PHA and then IFN-γ levels was compared in presence and absence of CsA [19,20].

For the cytokine neutralization assay, adjusted blood samples were treated with 1 pg/ml of cytokine neutralizing monoclonal antibodies composed of anti-human IL-12 (clone 24910.1, R&D system, USA), anti-IL-15 (clone34598.11, R&D system, USA), and anti-human IL-18 (clone 125-2H, Medical & Biological Laboratories, Japan) for 15 min before adding 18 HA units Den2. The cultured supernatants were harvested and kept at -20°C prior to IFN-γ detection by sandwich ELISA (BD OptEIA™ set Human IFN-γ, BD Bioscience, USA) as per manufacturer's protocol. The standard curve was linear up to 300 pg/ml and the detection limit was 18.8 pg/ml. Any cultured supernatants that showed an optical density higher than 300 pg/ml were diluted, retested and the IFN-γ concentration was estimated by multiplying with the dilution factor.

### Intracellular IFN-γ vs. cell surface markers staining and flow cytometric analysis

Whole blood samples containing 9 × 10^5 ^lymphocytes/ml were co-cultured with stimulators in FACS tubes (BD Bioscience) and 10 μg/ml brefeldin A (Sigma) was added for the last 3 h. Then, red blood cells in cultured samples were lysed by lysing solution (BD Bioscience, USA), and the samples washed once with 1% FCS-PBS, and Fc receptors were blocked with anti-CD16 for 30 min. Cultured cells were washed once and stained for cell surface markers using tri-color labeled anti-CD3 (Caltag Laboratories, USA) and PE-labeled anti-CD4 or PE-labeled anti-CD8 or PE-labeled-CD56 (PharMingen, USA) and FITC-labeled anti-CD4 or FITC-labeled anti-CD8 or FITC-labeled anti-CD16 (Caltag Laboratories). Isotype-matched immunoglobulin controls were included in each analysis. Staining of cell surface markers was performed for 30 min at 4°C, and the stained cells were washed twice in cold 1% FCS-PBS and fixed with 10% paraformaldehyde-PBS at 4°C for 15 min. Fixation was followed by permeabilization with 1% FCS-PBS, 0.1% saponin (Sigma) and 0.1% sodium azide (Sigma). Staining of intracellular IFN-γ was performed by incubating permeabilized cells with 1 μg/tube APC-labeled anti-human IFN-γ (Caltag Laboratories) in incubation buffer (1% FBS, 0.1% saponin and 0.1% sodium azide in PBS) at room temperature for 30 min. The stained cells were washed once and resuspended in 10% paraformaldehyde-PBS. Analysis was performed by FACSCalibur flow cytometer and CELLQuest software (BD Bioscience).

### Statistical analysis

The Mann-Whitney test and the Wilcoxon signed rank test for comparison and linear regression for correlation were performed using Graph Pad Prism software version 5 (GraphPad, San Diego, USA). A *P*-value less than 0.05 was considered statistically significant.

## Abbreviations

Den: dengue viral antigen; Den1, 2, 3 and 4: dengue viral serotype 1, 2, 3 and 4; IFN-γ: interferon-gamma; TCR: T cell receptor; CsA: cyclosporin A; IL: interleukin; DHF: dengue hemorrhagic fever.

## Authors' contributions

DS carried out the cellular studies and draft the manuscript. AR and CL participated in the design of the study. GL conceived of the study and participated in its design and coordination. All authors read and approved the final manuscript.

## Supplementary Material

Additional file 1 **Supplement figures Figure S1**: IFN-γ production of five healthy schoolchildren in responses to various stimulators. **Figure S2**: Titration of cyclosporin A (CsA) to inhibit IFN-γ induced by PHA. **Figure S3**: Titrations of anti-IL-12, anti-IL-15 and anti-IL-18 to decrease IFN-γ induced by heat killed *B. pseudomallei. ***Figure S4**. Calculation of % IFN-γ producing CD4^+ ^or CD8^+^T cells triggering via bystander and specific T cell activation in responses to Den2. **Figure S5**: IFN-γ induction by inactivated dengue virus serotypes 2 prepared from mouse brain extraction and cultured supernatants of C6/36 cell lines. **Figure S6**: Linear regression analysis of IFN-γ induction by inactivated dengue virus serotypes 2 antigens prepared from mouse brain extraction and cultured supernatants of C6/36 cell lines. **Figure S7**: Th1/Th2 cytokines induced by Den2. Click here for file
