# Supplementary material for: Bystander T cells in human immune responses to dengue antigens
Source: BMC Immunol. 2010 Sep 20;11:47. doi: 10.1186/1471-2172-11-47 (PMC2949776; doi:10.1186/1471-2172-11-47)
Supplement: Additional file 1 — Supplement figures Figure S1: IFN-γ production of five healthy schoolchildren in responses to various stimulators. Figure S2: Titration of cyclosporin A (CsA) to inhibit IFN-γ induced by PHA. Figure S3: Titrations of anti-IL-12, anti-IL-15 and anti-IL-18 to decrease IFN-γ induced by heat killed B. pseudomallei. Figure S4. Calculation of % IFN-γ producing CD4+ or CD8+T cells triggering via bystander and specific T cell activation in responses to Den2. Figure S5: IFN-γ induction by inactivated dengue virus serotypes 2 prepared from mouse brain extraction and cultured supernatants of C6/36 cell lines. Figure S6: Linear regression analysis of IFN-γ induction by inactivated dengue virus serotypes 2 antigens prepared from mouse brain extraction and cultured supernatants of C6/36 cell lines. Figure S7: Th1/Th2 cytokines induced by Den2. [file 1471-2172-11-47-S1.PDF]

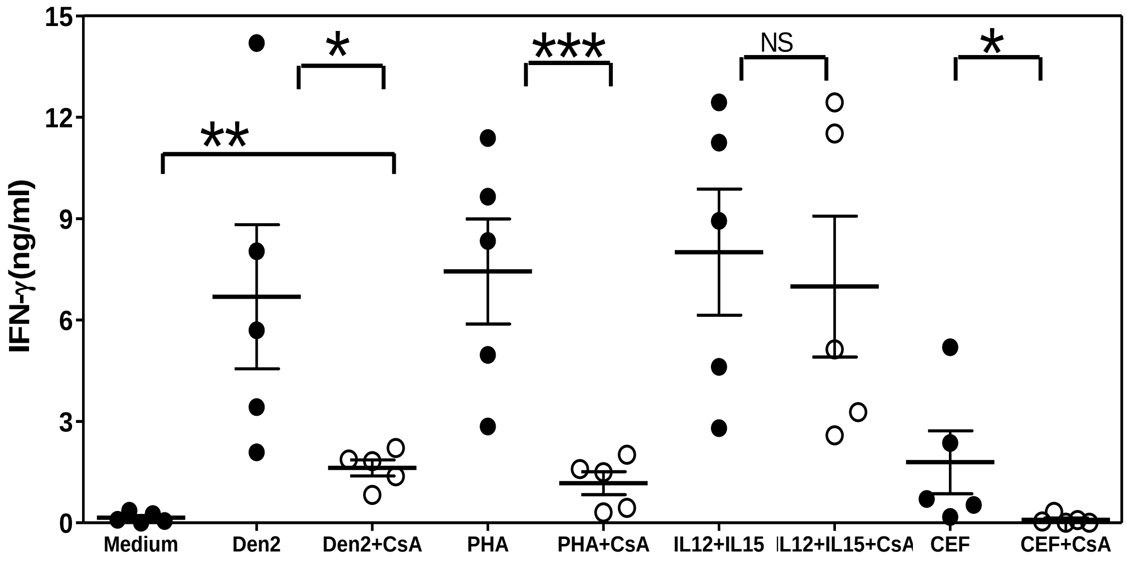

Figure S1: IFN-γ production of five healthy schoolchildren in responses to various stimulators. Whole blood samples were collected and stimulated with Den2, PHA, IL-12+IL-15 and a pooled peptides of Cytomegalovirus, Epstein Barr and Influenza viruses (CEF) in the condition with and without cyclosporin A (CsA) treatment for 48 h. IFN-γ production in cultured supernatants was examined by ELISA. *, ** or *** represents the statistically significantly difference of Mann-Whitney test between the two groups and p-value was less than 0.05, 0.001 and 0.0001, respectively. NS represents non-statistically significant difference.

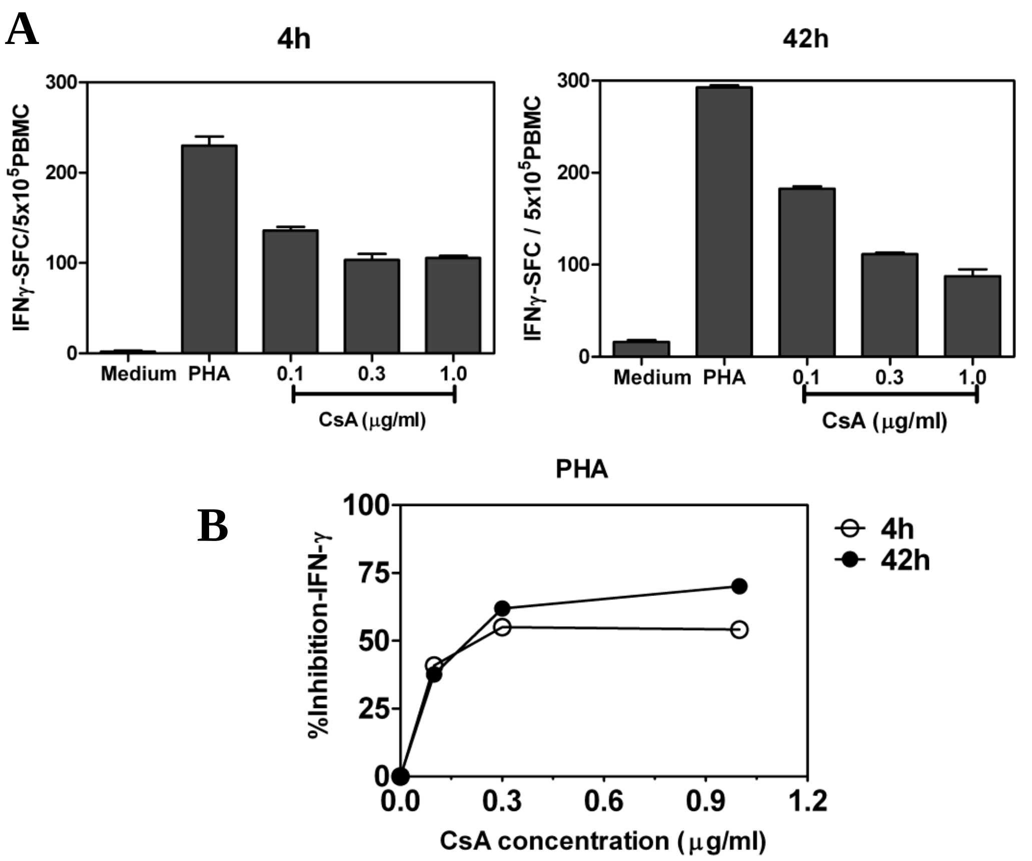

Figure S2: Titration of cyclosporin A (CsA) to inhibit IFN-γ induced by PHA. Whole blood samples were stimulated by PHA for 4 and 42 h (A) in the absence or presence of 0.1, 0.3 and 1 μg/ml of CsA. The IFN-γ levels were examined by ELISPOT and the % inhibition of IFN-γ production is shown (B).

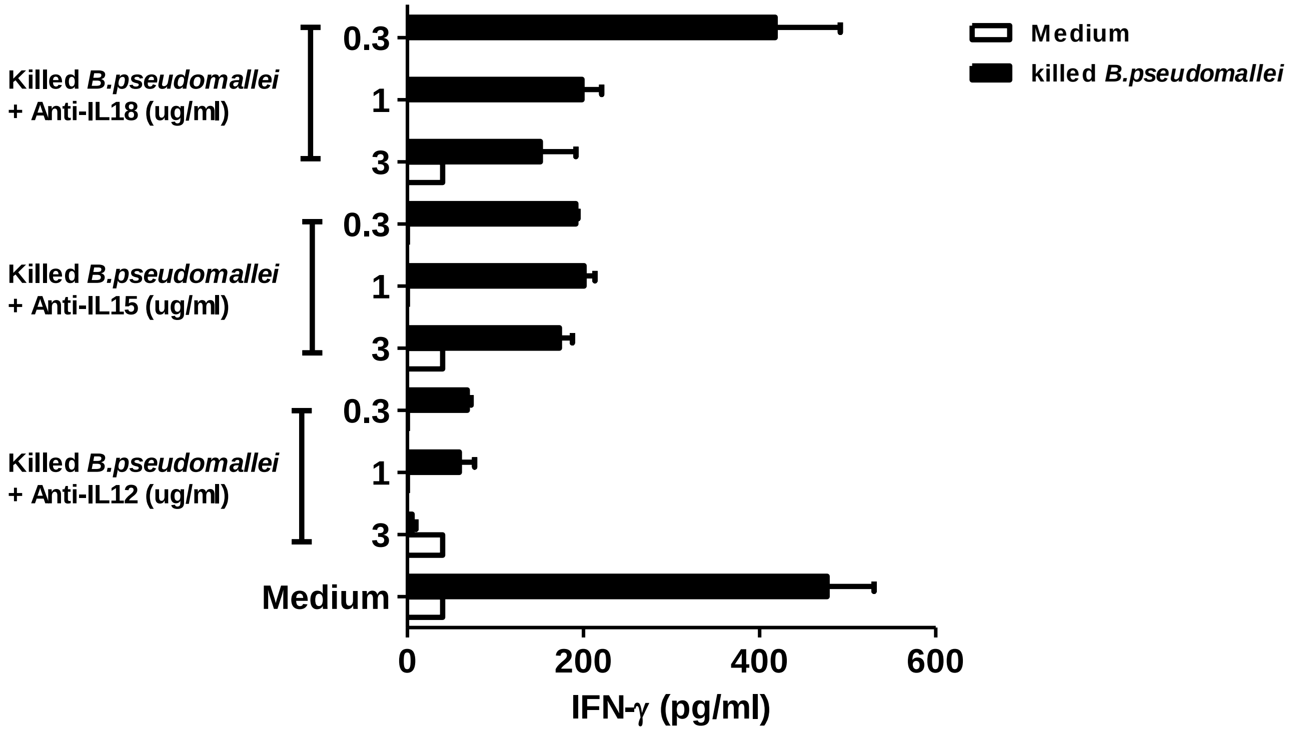

Figure S3: Titrations of anti-IL-12, anti-IL-15 and anti-IL-18 to decrease IFN-γ induced by heat killed *B. pseudomallei.* Whole blood samples containing 9×10^5 lymphocytes/ml were stimulated with heat inactivated *B. pseudomallei* at 3×10^7/ml in the presence of three-fold concentration of neutralizing antibodies to IL-12, IL-15 and IL-18 for 48 h. The cultured supernatants were examined for IFN-γ production by ELISA.

A

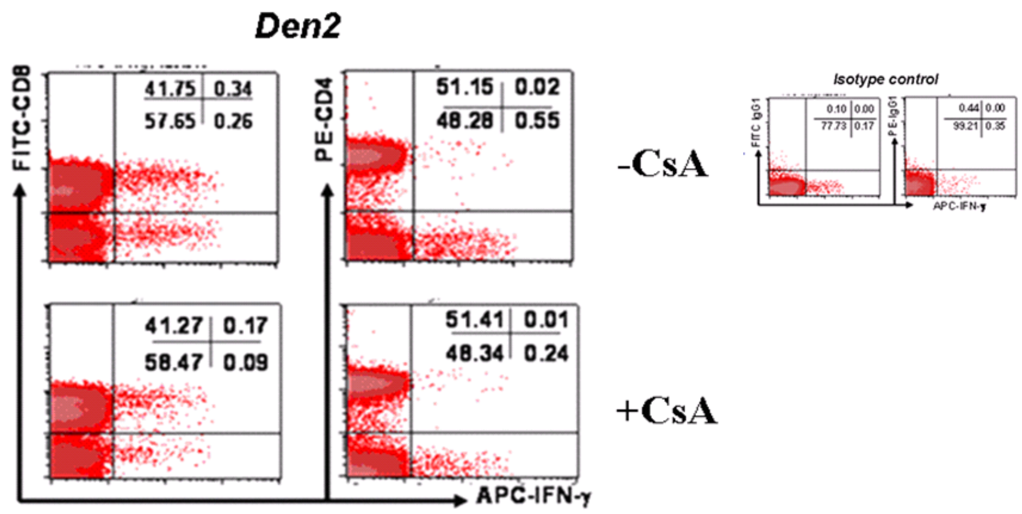

B

%IFN-γ producing CD4+ or CD8+T cells = $\dfrac{\text{UR X 100}}{\text{UL+ UR}}$

%IFN-γ derived bystander T cells = % IFN-γ resisted CsA (-CSA)

%IFN-γ derived specific T cells = %IFN-γ which was inhibited by CsA ((-CsA)- (+CsA)).

Figure S4: Calculation of %IFN-γ producing CD4+ or CD8+T cells triggering via bystander and specific T cell activation in responses to Den2. Whole blood samples were stimulated with Den2 in the absence (-CsA) or presence of 0.3 µg/ml of CsA (+CsA) for 24 h and the cultured cells were stained for surface markers vs. intracellular IFN-γ. Quadrant analysis of FITC-CD8 or PE-CD4 or isotype controls and APC-IFN-γ gating on $CD3^+$ small lymphocytes is shown (A). Calculation of %IFN-γ producing $CD4^+$ or $CD8^+$T cells triggering via TCR-specific and bystander is shown (B).

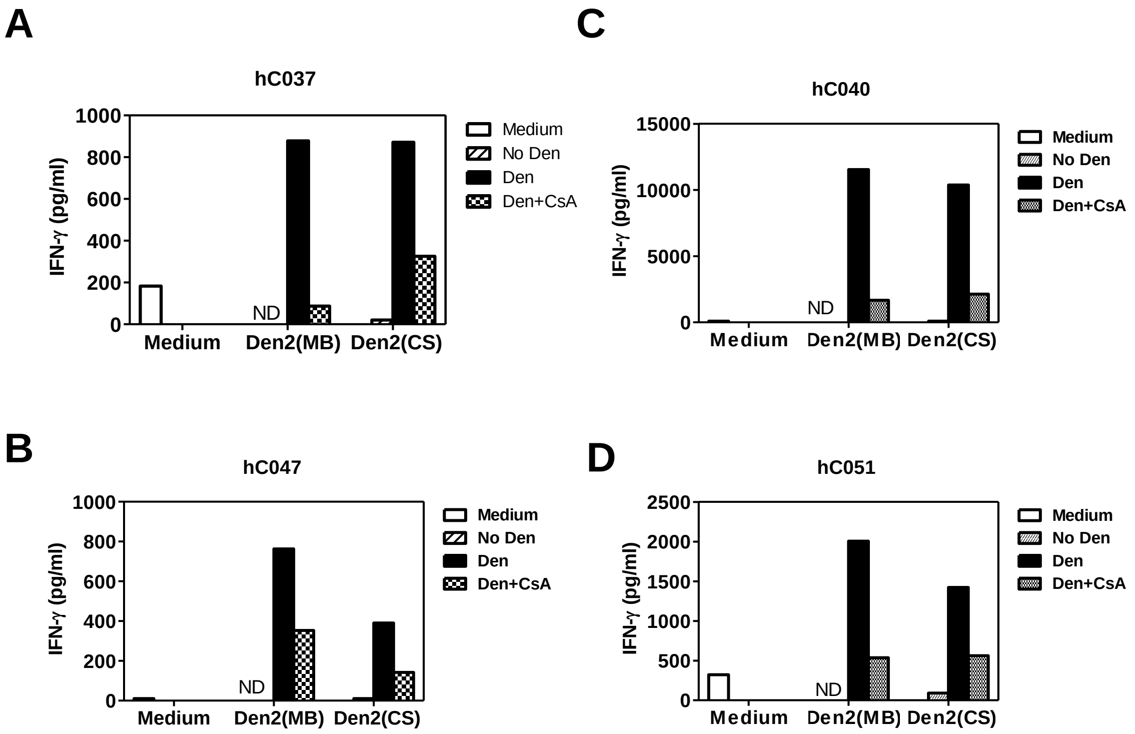

Figure S5: IFN-γ induction by inactivated dengue virus serotypes 2 prepared from mouse brain extraction (MB) and cultured supernatants of C6/36 cell lines (CS). Whole blood samples collected from four Thai school children were stimulated with two preparation of inactivated Den2 from mouse brain extracted (MB) antigen and Den2 infected culture supernatants of C6/36 cell line (CS), details as described in Figure 1 and the negative control of cultured supernatants from uninfected C6/36 cell line (no Den) was included. The IFN-γ production of four children is individually shown (A-D). ND represents not determined.

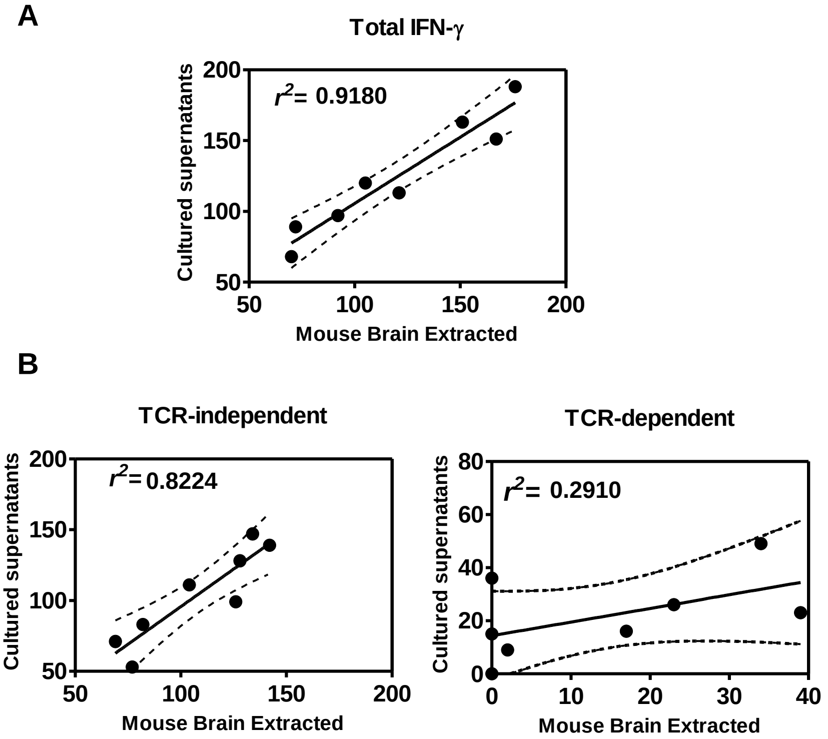

Figure S6: Linear regression analysis of IFN-γ induction by inactivated dengue virus serotypes 2 antigens prepared from mouse brain extraction (MB) and cultured supernatants of C6/36 cell lines (CS). Whole blood samples collected from 8 healthy donors were stimulated with the two preparations of inactivated Den2 including mouse brain extract (MB) and cultured supernatants of C6/36 cell line (CS) in the presence and absence of CsA. Total IFN-γ production (A), IFN-γ induction via bystander (TCR-independent) and via TCR-dependent activation (B), r^2 represents correlation coefficient.

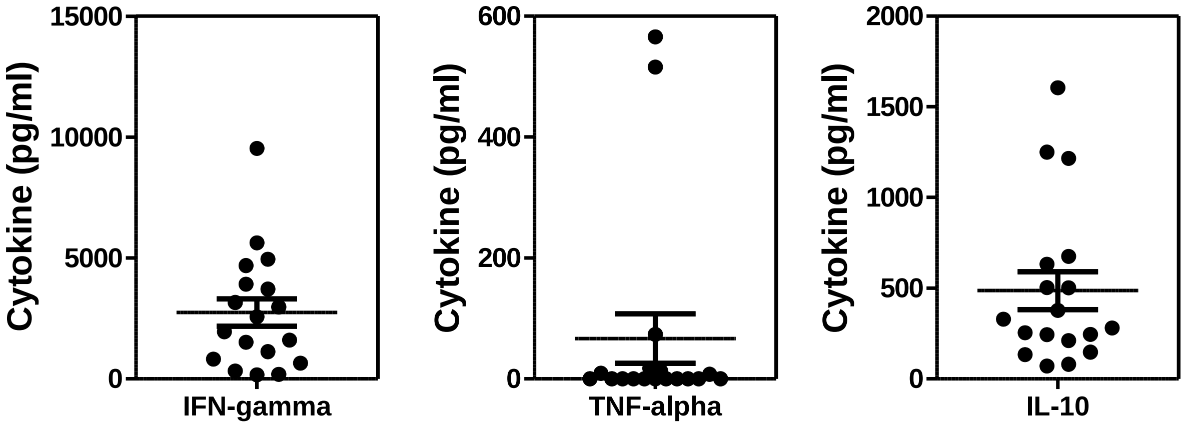

Figure S7: Th1/Th2 cytokines induced by Den2. Whole blood samples collected from 18 children were stimulated with Den2 for 48 h and cytokines in cultured supernatants were assayed by cytometric bead array.
